# Parent-rated behavior problems associated with overweight before and after controlling for sleep disordered breathing

**DOI:** 10.1186/1471-2431-6-34

**Published:** 2006-12-14

**Authors:** Shelagh A Mulvaney, Kristine L Kaemingk, James L Goodwin, Stuart F Quan

**Affiliations:** 1 School of Nursing and Department of Pediatrics, Vanderbilt University, Nashville, Tennessee, USA; 2 Department of Pediatrics, University of Arizona, College of Medicine, Tucson, Arizona, USA; 3 Arizona Respiratory Center, University of Arizona, College of Medicine, Tucson, Arizona, USA

## Abstract

**Background:**

Researchers and clinicians are seeking to develop efficacious behavioral interventions to treat overweight children; however, few studies have documented the behavioral correlates of overweight children in community samples. The goal of this study was to determine the nature and prevalence of behavior problems for overweight school-aged children versus normal weight peers before and after controlling for the effect of sleep disordered breathing.

**Methods:**

Hispanic and Caucasian children were invited to participate in a study of sleep through public elementary school classrooms. Anthropometric evaluation and behavioral ratings were collected for 402 children aged 6–11 years. Overweight was calculated using the Centers for Disease Control age- and gender-specific guidelines. Children were classified as overweight if they were at or above the 95th percentile for their age and gender group. Behavior problems were measured using the Conners' Parent Rating Scales-Revised and the Child Behavior Checklist. Sleep disordered breathing was assessed using in-home overnight polysomnography.

**Results:**

Approximately 15% (59/402) of the sample was classified as overweight. Simple odds ratios indicated that overweight children were more likely to have clinically relevant levels of internalizing symptoms (OR 2.23, CI 1.05–4.72), psychosomatic complaints (OR 2.15, CI 1.02–4.54), withdrawal (OR 4.69, CI 2.05–10.73), and social problems (3.18, 1.53–6.60). When odds ratios were adjusted for level of sleep disordered breathing, withdrawal (OR 3.83 CI 1.59–9.22) and social problems (OR 2.49 CI 1.14–5.44) remained significantly higher for overweight subjects.

**Conclusion:**

After controlling for the effect of sleep disordered breathing, behaviors such as withdrawal and social problems, are common in overweight children and need to be taken into account in the design of interventions and services as they may act to moderate the efficacy of behavioral treatments.

## Background

The prevalence of childhood overweight has been reported frequently in the research literature and popular media. Recent epidemiological studies estimate that pediatric overweight has increased dramatically in the last generation, that as of 2000 approximately 15% of children aged 6–11 are overweight [1], and that 25–31% of children and adolescents in the U.S. are overweight or at risk of being overweight [2-5]. The health consequences related to childhood overweight include insulin resistance, type 2 diabetes, hypertension, and heart disease later in life [6,7].

Psychological or behavioral problems in childhood have been examined as both causes and effects of overweight. That is, overweight has been hypothesized as a possible result of psychological symptoms and psychological symptoms have been hypothesized to be a result of overweight [8-11]. Further evidence for the relationship between overweight and behavioral problems is provided by treatment studies that have shown decreased levels of psychological and behavioral problems in children subsequent to treatment for overweight [12-14].

Clinical, referred, or screened samples have found relationships between overweight and depression [15,16], social problems, withdrawal [15], and both internalizing and externalizing behaviors [16]. One longitudinal study found relationships between chronic overweight and oppositional defiant disorder for boys and girls and with depression for boys [10]. Alternatively, an epidemiological study of adolescents and young adults found a relationship between body mass index (BMI) and depression only in girls [16]. Mixed samples have found lowered social and physical perceived self-competence and well-being compared to normal weighted peers [17]. Community-based studies have found depression [18] and overweight related in girls and non-specific patterns of behavior problems associated with overweight [8]. In overweight children and adolescents, quality of life measures indicate a lower overall quality of life as well as lower self-esteem and physical functioning [19,20].

Sleep disordered breathing and obesity are comorbid in adults [21]. There is some support for this relationship in children. Children diagnosed with sleep disordered breathing (SDB) may have a greater probability of comorbid overweight [22] and children who are overweight have been found to have a greater probability of SDB [23]. However, in previously published analyses, the TuCASA study (from which the current analyses are derived), have not confirmed this relationship [24].

There are several limitations to the studies currently available linking overweight to behavior in children. Studies using non-referred samples have been screened for specific psychological disorders [10], are conducted within other cultures [17], or have used overweight categorizations that are not age- and gender-specific [15,16]. While behavioral problems have been linked to SDB, such as cognitive problems, hyperactivity, and externalizing behaviors [25-27] and behavior problems have been linked to overweight (as described above), ideally weight and SDB should be considered simultaneously in estimating their relationships with behavior.

Identification of behaviors uniquely related to obesity could inform development of behavioral interventions and services for children. Clinicians working in this area could benefit from understanding the independent contribution of overweight on behavioral problems both in the presence and absence of SDB. Thus, the goal of the current research was to provide a description of the nature and prevalence rate of behavior problems, in a general population cohort, for overweight and normal weight school children after controlling for the presence of sleep disordered breathing. We hypothesized that externalizing behaviors would be less related to overweight after taking into account levels of SDB.

## Methods

### Participants

Subjects in the current analyses were enrolled in a study of sleep in children, the Tucson Children's Assessment of Sleep Apnea [24,27,28]. This study recruited 6 through 11 year old Hispanic and Caucasian children to undergo home polysomnography, a sleep questionnaire, and neurocognitive testing. A detailed description of recruitment procedures has been previously published [24,27,28]. Briefly, recruitment was accomplished by soliciting the cooperation of 19 selected elementary schools in the Tucson Unified School District (TUSD). A short sleep-habits screening questionnaire was sent home with all children in a "notes home" folder. Parents were asked to complete the questionnaire and to provide contact information if they agreed to allow study personnel to call and schedule a polysomnogram and neurocognitive testing for their child. Sleep and demographic questionnaire data, child weight and height measures were acquired in the family home at the time of the polysomnogram. Behavioral questionnaire data were collected from parents when the child subsequently underwent neurocognitive testing within the Department of Pediatrics. The TUSD Research Committee and the University of Arizona Institutional Review Board approved the study protocol. Parents or guardians completed approved consent forms and children completed assent forms before participating in the study.

Children were excluded from the study if there was a history of head injury, tonsillectomy, mental retardation, or asthma. Families were paid $25 for completing the sleep study and $25 for completing the behavioral evaluation.

### Measurement

#### Weight

Children were classified as overweight if their BMI was at or over the 95th percentile for age- and gender-specific normative values [29]. Children below the 95th percentile were classified as "normal weight". The group referred to as "normal weight" included those children at risk for overweight (between the 85th and 94th percentiles for their sex and age).

#### Behavior

The Conners' Parent Rating Scale – Revised (CPRS-R) is a well validated 80-item behavior rating scale that measures symptoms of attention deficit hyperactivity disorder (ADHD; hyperactivity, impulsivity, and inattention) as well as comorbid behaviors such as oppositional behavior, anxiety, and somatic complaints [30]. All 12 CPRS-R scales focus on behaviors central to a diagnosis of ADHD such as Cognitive Problems and Hyperactivity or measure behaviors that are commonly comorbid with inattention and hyperactivity, such as social problems. Three scales on the CPRS-R are considered internalizing correlates of ADHD (Anxious-Shy, Perfectionism, and Psychosomatic Complaints). Seven of the scales on the CPRS-R are derived directly from the Diagnostic and Statistical Manual-IV criteria for ADHD [31]. Behaviors are rated on a 4-point scale that ranges from 'Very True' to 'Not True'. A t-score is derived for each scale, based on a large age and gender specific normative sample. A t-score (M = 50, SD = 10) over 65 is considered to indicate moderate to severe clinical impairment.

The Child Behavior Checklist (CBCL) [32] allows assessment of 118 parent-reported behavioral and emotional problems of children aged 4–18. Parents rate their child's behavior on a 3-point scale (Not True, Somewhat True, or Very/Often True). The CBCL includes 8 syndrome scales, a Total problem score, and higher order Internalizing and Externalizing scales. Internalizing scales include Anxious/Depressed, Withdrawn, and Somatic Complaints. Externalizing scales include Aggressive Behavior and Delinquent Behavior. Three syndrome scales, Social Problems, Thought Problems, and Attention Problems, are not part of the internalizing/externalizing dimensions. A t-score over 65 was considered to indicate moderate to severe clinical impairment.

#### Polysomnography

Home visit procedures and methods for obtaining PSG data have been described previously. Briefly, a 2-person mixed-gender team arrived at the home approximately 1 hour prior to the child's typical bedtime. Institutional Review Board-approved informed consent was obtained from the parent, and an assent form was signed by the child. Questionnaires were administered, and anthropometric and other physiologic measurements were completed. The oral airway was examined by a trained technician and rated with a value from 1 (unobstructed) to 3 (tonsils encroaching upon airway). Unattended overnight polysomnograms were obtained using the Compumedics PS-2 system (Abbotsford, Victoria, Australia). The following signals were acquired as part of the TuCASA montage: C_3_/A_2 _and C_4_/A_1 _electroencephalogram, right and left electrooculogram, a bipolar submental electromyogram, thoracic and abdominal displacement (inductive plethysmography bands), airflow (nasal/oral thermister), nasal pressure cannula, finger pulse oximetry, electrocardiogram to detect major arrhythmias (single bipolar lead), snoring microphone, body position (mercury gauge sensor), and ambient light to determine sleep period time (sensor attached to the vest to record on/off).

The Compumedics software system was used to process all PSG data. Scoring has been described in detail previously [28]. Briefly, sleep stages were scored according to Rechtschaffen and Kales criteria. Arousals were identified using criteria published by the American Academy of Sleep Medicine. Apneas were scored if the amplitude (peak to trough) of the airflow signal using the thermister decreased below at least 25% of the amplitude of baseline breathing (identified during a period of regular breathing with stable oxygen levels), if this change lasted for more than 6 seconds or 2 breath cycles. Hypopneas were designated if the amplitude of any respiratory signal decreased below (approximately) 70% of the amplitude of baseline and if the thermister signal did not meet the criterion for apnea. Central events were marked if no displacement was noted on both the chest and abdominal inductance channels. Otherwise, events were scored as obstructive. After full scoring, analysis software was used to link each event to data from the oxygen saturation and arousals from the electroencephalogram channels.

The Respiratory Disturbance Index (RDI) was used as an indicator of SDB and defined as the number of respiratory events (apneas and hypopneas) per hour of the total sleep time. Breathing "events" were scored during sleep if total inspiratory volume went below 50% of baseline and was associated with a 3% decrease in blood oxygen levels [28,33,34]. For this analysis, a 3% oxygen desaturation was required for an event to be counted in the total RDI. We considered a child to have SDB if their RDI was greater than or equal to 1 event per hour of total sleep time. Use of this definition is supported by previous evidence that an RDI of one, based on events with a 3% oxygen desaturation, is clinically significant [35].

### Data analyses

Data were analyzed using SPSS version 13 for Windows (SPSS, Inc., Chicago, IL). One-way ANOVA or Chi2 tests were used to test differences in demographic statistics between overweight and normal weight children. Simple odds ratios and adjusted odds ratios were calculated to determine the probability of behavior problems given the presence of overweight using the behavioral measures CBCL and CPRS-R. Adjusted odds ratios were calculated for the behavioral measures and controlled for the level of sleep disordered breathing using the respiratory disturbance index (RDI), described above.

## Results

Of the 7055 initial surveys distributed throughout the schools, 2327 (32.9%) were returned. Of the 1219 (52.4%) who gave permission to be contacted further, 503 (41.3%) met inclusion criteria and agreed to participate. Of those 503, 480 had sleep studies of sufficient quality. Of the 480 sleep studies completed, 402 (83.7%) had complete anthropometric and behavioral data.

Descriptive summary statistics for the sample are in Table 1. There were approximately equal numbers of boys and girls, and more Caucasians than Hispanics. Overweight was present in 14.7% of the total sample. There were significantly more Hispanic subjects in the overweight (55.9%) group compared to the non-overweight group (35.6%). Descriptive statistics are provided separately for the overweight and normal weight subjects in Table 2. The overweight subjects tended to be slightly older than the normal weight subjects. Although there was a trend toward gender differences, there were no significant differences between the groups on RDI, gender, or parent education.

**Table 1:** Descriptive statistics for the sample (N = 402).

Variable	Mean (SD) or Percent	Range
Age (years)	8.8 (1.61)	6.0 – 11.9
Gender (female)	48.3 %	
Hispanic	38.6 %	
Caucasian	61.4 %	
Overweight	14.7 %	
BMI	18.0 (4.48)	10.9 – 48.1
Parent Education*	13.6 (3.24)	1–21

*(N = 287) Many parents did not report educational level.

**Table 2:** Descriptive statistics and tests of differences between normal weight versus overweight subjects.

	Normal Weight Mean (SD) or Percent (N = 343)	Overweight Mean (SD) or Percent (N = 59)	F (sig) or Chi^2 ^(sig)
Age	8.7 (1.6)	9.2 (1.5)	F 4.42 (.04)
Gender (% Female)	50.1%	37.3%	Chi 3.33 (.07)
Hispanic %	35.6%	55.9 %	Chi 8.81 (.00)
RDI*	0.92 (2.15)	0.81 (1.21)	F 0.19 (.66)
Parent Education	13.7 (3.3)	13.2 (2.8)	F 0.90 (.34)

*Respiratory Disturbance Index

Table 3 shows percentages of overweight and normal weight subjects who were classified as having clinically relevant levels of behavioral problems (moderate to severe) for the Conners' Parent Rating Scale-Revised (CPRS-R). Table 3 includes simple odds ratios indicating the probability of overweight children being classified with clinically relevant levels of behavior problems. Odds ratios, adjusted for SDB, are also shown in Table 3. Although odds ratios were significant for several CPRS-R scales, confidence intervals were wide and only the Psychosomatic scale was statistically significant. Twice as many children were classified as having psychosomatic complaints in the overweight versus normal weight groups. When odds ratios were adjusted for level of SDB, psychosomatic complaints were no longer significantly different across the groups.

**Table 3:** Odds ratios and percentages for probability that a subject who is overweight will be classified within the clinical range for each problem behavior on the CPRS-R.

**Scale name**	% Normal Weight in Clinical Range	% Overweight in Clinical Range	Unadjusted Odds Ratio (95% CI)	Adjusted Odds Ratio* (95% CI)
Oppositional	9.4	16.7	1.69 (0.76–3.74)	1.62 (0.71–3.72)
Cognitive Problems	11.7	20.0	1.68 (0.80–3.49)	1.46 (0.68–3.16)
Hyperactivity	17.3	16.7	0.84 (0.39–1.82)	0.86 (0.39–1.88)
Anxious Shy	12.3	15.0	1.29 (0.59–2.81)	1.21 (0.54–2.72)
Perfectionism	6.4	5.0	0.78 (0.22–2.69)	0.68 (0.18–2.49)
Social Problems	8.5	15.0	1.63 (0.71–3.76)	1.26 (0.51–3.09)
Psychosomatic	9.4	20.0	**2.15 (1.02–4.54)**	1.59 (0.71–3.56)
ADHD index	12.0	11.7	0.81 (0.32–2.00)	0.64 (0.24–1.69)
Global Index- Total	12.3	10.2	0.65 (0.2–41.73)	0.62 (0.23–1.69)
DSM Inattentive	12.3	16.7	1.25 (0.57–2.73)	1.05 (0.46–2.40)
DSM Hyperactive	17.6	18.3	0.94 (0.45–1.95)	0.98 (0.46–2.09)
DSM Total	14.4	13.3	0.78 (0.33–1.82)	0.67 (0.27–1.63)

* Note: Odds Ratios controlled for level of sleep disordered breathing.

Table 4 shows percentages of overweight and normal weight subjects who were classified as having clinically relevant levels of behavioral problems (moderate to severe) for the Child Behavior Checklist, simple odds ratios, and adjusted odds ratios controlling for SDB. Significant unadjusted odds ratios were observed for the Withdrawn, Social Problems and Internalizing scales. When SDB was controlled, the Withdrawn (3.83, CI 1.59–9.22) and Social Problems (2.49, CI 1.14–5.44) odds ratios remained significantly different between the groups.

**Table 4:** Percentages, unadjusted, and adjusted odds ratios for probability that a subject who is overweight will be classified within the clinical range for each problem behavior on the CBCL.

Scale name	% Normal Weight in Clinical Range	% Overweight in Clinical Range	Unadjusted Odds Ratio (95% CI)	Adjusted Odds Ratio* (95% CI)
Aggressive Behavior	7.4	10.2	1.12 (0.41–3.06)	0.96 (0.33–2.79)
Anxious/Depressed	9.5	16.9	1.69 (0.76–3.75)	1.46 (0.63–3.40)
Attention Problems	14.0	11.9	0.69 (0.28–1.07)	0.54 (0.20–1.42)
Somatic Complaints	10.1	16.9	1.58 (0.72–3.50)	1.21 (0.51–2.85)
Withdrawn	4.5	20.3	**4.69 (2.05–10.73)**	**3.83 (1.59–9.22)**
Social Problems	8.0	23.7	**3.18 (1.53–6.60)**	**2.49 (1.14–5.44)**
Thought Problems	9.2	8.5	0.70 (0.24–2.07)	0.57 (0.18–1.81)
Delinquent Behavior	11.3	10.2	0.72 (0.27–1.91)	0.73 (0.26–1.98)
Total Score	12.2	18.6	1.46 (0.68–3.11)	1.38 (0.63–3.03)
Internalizing	9.2	20.3	2.23 (1.05–4.72)	1.84 (0.83–4.10)
Externalizing	7.8	10.2	1.08 (0.39–2.92)	0.92 (0.32–2.65)

* Note: Odds Ratios controlled for level of sleep disordered breathing.

## Discussion

Within this sample, 15% of the subjects were overweight. Overweight was more prevalent in Hispanic and male subjects. Overweight children had increased parent reports of psychosomatic complaints, social problems, withdrawal, and general internalizing behaviors. When SDB was taken into account, overweight was no longer associated with psychosomatic complaints and general internalizing symptoms. However, levels of withdrawal for the overweight subjects were still almost 4 times higher and social problems were 2.5 times higher than that of normal weight subjects. Hyperactivity, oppositional, and externalizing behaviors were not elevated in the overweight group and showed minimal change after accounting for SDB.

While both obesity and SDB may have behavioral consequences, each may be related to different types of behavioral problems and each may affect behavior through unique causal mechanisms. For example, SDB may cause externalizing types of behavior problems through associated nocturnal hypoxia and disrupted sleep architecture. Reactions to and maladaptive coping with functional limitations and social stigma may moderate the relationship between overweight and behavior problems. The Conners' psychosomatic complaints scale, which was related to overweight before but not after controlling for SDB, included items related to headaches, stomach aches in general, stomach aches before school, vague complaints that are not supported by physical illness, and fatigue. While in overweight children these behaviors may be more related to avoidance of school or other social situations, vague bodily pain, malaise and fatigue could also be related to poor quantity or quality sleep associated with sleep disordered breathing.

The most prevalent problem behaviors reported by parents on the CBCL were those related to social problems and withdrawal. The CBCL withdrawn scale includes items related to shyness, preference for being alone, secretiveness, sulking, underactive, sadness, withdrawal, and not talking. Although overweight has been hypothesized to be a result of some psychiatric symptoms [8], these behavioral problems could result from living with overweight. Social problems on the CBCL were reported much more frequently by parents of overweight children than by parents of normal weight children. Social problems scale items are related to immaturity, not being liked by peers, teased by peers, overweight, and clumsiness. Children who are overweight may be subject to bullying or face functional limitations [36]. They may be ostracized by their peers or feel less physically competent compared to their peers [17]. Withdrawal may be a reaction to judgment by peers or other social problems.

Oppositional behavior has been linked to chronic overweight, when overweight is present from childhood through adolescence [11]. The current results do not support the idea that oppositional or externalizing behaviors, such as aggression, are salient at this developmental stage in the context of overweight. Another large cross-sectional study that found a relationship between overweight and overall behavior problems did not fit specific patterns related to externalizing (nor internalizing) problems [8]. It may be that the current sample was not chronic enough to be at risk for externalizing behaviors.

A limitation to this study is that only one informant was used to measure behavioral problems. Although cross-informant behavioral agreement is generally low [37], it does provide contextual perspectives on behavior and may be used to generate alternate explanations for results. Additionally, this study is descriptive and the data do not allow for strong inferences regarding the etiology of the behaviors reported by parents. Finally, more data on these families would have been helpful to determine if familial nutrition habits, parental obesity, and/or socioeconomic status related to behavior problems. However, this study did examine a broad variety of problem behaviors derived from a population-based sample of school children. Overweight was defined as age- and gender-specific, however, the overweight group was predominantly Hispanic. Epidemiological studies have documented the relatively high rate of overweight in young Hispanic males found here [3].

Overweight may contribute to behavior problems independent of SDB. It is possible that the behaviors for which overweight adds predictive value above SDB are those that are influenced by social factors such as teasing or other peer behaviors. Overweight children do face discrimination and stigmatization and this may impact their global self-worth [38-40]. Further research with larger samples should examine the extent to which emotional response to overweight is moderated by environmental and social pressures such as exclusion or individual differences in the need for relatedness or social acceptance.

The current cross- sectional study allows limited causal inference. As Freidman and Brownell [41] have emphasized in adults, many important questions about etiology and treatment may only be answered through longitudinal research that allows for an examination of multiple risk factors. Although emotional and behavioral problems may influence the efficacy of interventions in obesity, many trials to date have not measured or examined the moderating effect of behavioral problems on outcomes. The current study indicates that behaviors associated with overweight, such as withdrawal and social problems, may need to be taken into account in the design of interventions and services as they may act to moderate the efficacy of behavioral treatments. Additionally, the predisposition toward behavioral problems in the context of childhood overweight may be influenced by long-term attitudes surrounding overweight and the extent of obesity within the family. Psychologists and health administrators seeking to create services responsive to the rates in U.S. childhood overweight could benefit from further information regarding the psychosocial mechanisms of any behavioral problems.

## Conclusion

In conclusion, after controlling for the effect of sleep disordered breathing, behaviors such as withdrawal and social problems, were present in some overweight children and should be considered in the design of interventions and services as they may act to moderate the efficacy of behavioral treatments for obesity.

## Abbreviations

SDB, sleep disordered breathing; RDI, respiratory disturbance index; CBCL Child Behavior Checklist; CPRS-R, Conners' Parent Rating Scale – Revised.

## Competing interests

The author(s) declare that they have no competing interests.

## Authors' contributions

SM conceived the manuscript, performed statistical analyses, helped with behavioral data collection, and drafted the manuscript. KK participated in the design and funding of the study, supervised behavioral data collection, and helped edit the manuscript. JG supervised PSG data collection, and edited the manuscript. SQ obtained funding for the project, designed the sleep study, and helped draft the manuscript. All authors read and approved the final manuscript.

## Pre-publication history

The pre-publication history for this paper can be accessed here:


